# Mortality From Combined Fractures of the Atlas (C1) and Axis (C2) in Adults

**DOI:** 10.7759/cureus.27554

**Published:** 2022-08-01

**Authors:** Fraser I Riddoch, Anna Leerssen, Rashid Abu-Rajab, Andraay Leung

**Affiliations:** 1 Trauma & Orthopaedics, NHS Greater Glasgow & Clyde, Glasgow, GBR; 2 General Practice, Royal Alexandra Hospital, Glasgow, GBR; 3 Trauma & Orthopaedics, Royal Alexandra Hospital, Glasgow, GBR; 4 Spinal Surgery, Queen Elizabeth Hospital, Birmingham, GBR

**Keywords:** cervical spine, atlas, axis, survivorship, morbidity and mortality

## Abstract

Study design

A retrospective case report of all upper cervical spine fractures diagnosed by CT imaging between 01/01/2013 and 31/12/2015 in NHS Greater Glasgow and Clyde, Scotland.

Objective

To compare the mortality following combined fractures of the atlas and axis to that of isolated fractures of either vertebra.

Background

The mortality from axis fractures is well documented in the literature. However, a combined fracture of the atlas and axis is seldom reported, leading to relatively unknown outcomes and mortality.

Methods

A total of 171 patients with atlas and/or axis fractures. Thirty-three presented with concurrent lower cervical spine fractures and were excluded from further analysis. Kaplan-Meier curves were used to compare survivorship between 108 patients with isolated and 30 with combined fractures. Similar analysis adjusted for comorbidities, including dementia and previous fragility fractures.

Results

Patients were followed up for 47.3±10.3 months (SD). Patients with isolated atlas fractures were significantly younger than those with an axis or combined fracture. Nearly half (8/17) of combined fracture mortalities occurred within the first 120 days. The mortality at 120 days was 26.7% in the combined fractures group and 18.5% in the isolated fracture group. There was no significant difference in the 120-day and overall mortality between these injury patterns. Furthermore, cognitive impairment and previous fragility fractures bore no significant impact on mortality. Nevertheless, mortality in the combined fracture group with previous fragility fractures did trend to shorter survivorship.

Conclusions

Patients with combined fractures are older and with the ever-increasing elderly population, the incidence of these injuries is expected to rise. While our data show that the 120-day mortality is proportionally higher in the combined fractures group, no long-term statistically significant difference is demonstrated. This evidence contests the notion that combined fractures of the atlas and axis have higher mortality than isolated injuries of either cervical vertebra.

## Introduction

Owing to the unique anatomy and biomechanical relationship of the atlas (C1) and axis (C2) vertebrae, trauma to the atlantoaxial complex can produce a wide array of injury patterns. Sir Geoffrey Jefferson’s historic case series of 46 atlas fractures, from 1920, was one of the first to describe combined fractures of the atlas and axis. He noted that 19 cases had this injury pattern and that these patients suffered an increase in morbidity and mortality [1]. The incidence of such combined injuries has been documented in the recent literature: 5-53% of patients with Type II or III odontoid fractures and 6-26% of patients with Hangman’s fractures have a concurrent atlas fracture while 24-53% of patients with an atlas fracture have been demonstrated to also have an odontoid fracture [2].

Several authors have concluded patients with combined upper cervical spine fractures have a higher mortality rate [3-6]. Fowler et al. observed that six of seven patients died in the early treatment period and Hanssen and Cabanela commented on five of six patients who died within the first 40 days of injury [4,6]. However, many such studies are small, from singular, specialist centres and now nearly three decades old.

On their own, atlas and axis fractures have two key causes - high-energy trauma in the young and falls in the osteoporotic elderly [7-8]. With the elderly proportion of the developed world’s population increasing, it has been predicted that the incidence of fractures in the upper cervical spine will also rise [9-10]. This presents new and interesting questions pertaining to mortality following such an injury. With the hypothesised increasing age of these patients, will this and other comorbidities negatively impact survival following upper cervical spine fractures? And can these patient factors be offset by the continuously developing world of preventative medicine, diagnostics and healthcare?

The aim of this study was to compare the mortality following combined fractures of the atlas and axis to that of isolated fractures of either cervical vertebra in adults. A secondary aim was to identify features in a patient’s past medical history, which would predispose them to shorter survivorship following such an injury.

## Materials and methods

All cervical spine computed tomography (CT) scans performed between 01/01/2013 and 31/12/2015 in NHS Greater Glasgow and Clyde, Scotland, United Kingdom were identified using Carestream’s Patient Archiving and Communications System (PACS). NHS Greater Glasgow and Clyde is the sole public health provider in the city of Glasgow, serving a population of around 1.2 million as part of the universally free healthcare system in the United Kingdom [11]. The formal radiology reports were reviewed and used to identify patients with fractures of the atlas and/or axis. All patients with concurrent sub-axial cervical spine fractures were excluded from further analysis. This allowed for the comparison of patients with cervical spine fractures isolated to the atlas, axis or both vertebrae.

Over the reviewed three-year period, there were 171 patients with CT-diagnosed fractures of their upper cervical spine. Of these, 33 patients had concurrent fractures in their lower cervical spine and were subsequently excluded. Scans performed in the Institute of Neurosciences were also excluded from this review, as these were often tertiary referrals, from outwith the health board, to the Scottish national specialist centre for spinal injuries.

Online patient records and scanned medical notes were reviewed for each case. General epidemiological data and medical comorbidities, including previous fragility fractures and cognitive impairment, were collected for all. Similarly, the mechanism of injury, neurological deficit, acute length of stay, treatment modality and outcomes from follow-up clinic letters were logged.

Statistical analysis was undertaken using SPSS v. 25 (IBM Statistics, IBM Corp., Armonk, NY). For analysis, the 138 included patients were divided into three cohorts: group 1 had isolated fracture(s) of the atlas (n=17), group 2 with isolated fracture(s) of the axis (n=91) and group 3 were those patients with fractures of both vertebrae (n=30). Kaplan-Meier survivorship curves were constructed and compared using the log-rank test. Categorical data were compared using the chi-squared and Mann-Whitney U tests. Statistical significance was set at p ≤ 0.05.

## Results

Patients were followed up on average for 47.3 ± 10.3 months (SD). There were 54 men and 84 women. The male-to-female ratio was 1:1.56. The mean age of patients with an isolated atlas fracture(s) was 58.7 years; 73.9 years for those with an isolated axis fracture(s) and 81 years for patients with combined fractures. Patients with combined fractures were significantly older (mean 80.97 ± 9.61 (SD)) than patients with an isolated fracture to either vertebra (71.45 ± 18.67 (SD)) (p = 0.011). One-way analysis of variance (ANOVA) (F=9.7, p<0.001) showed a significant difference in age between patients with a C1 fracture and C2 fracture and combined (p=0.001 and p<0.001, respectively) but not between patients with C2 and combined fractures (p=0.251).

A low-velocity fall from standing or sitting was the most common cause of injury, occurring in 87 (64%) of all patients. Table 1 describes the mechanisms of injury for each patient group. For analysis purposes, “high energy” mechanisms included road traffic collisions, falls from height and sporting injuries. A small proportion of mechanisms were recorded as “other,” as patients either presented several weeks after a possible injury or could not recall any trauma. Patients sustaining their fracture(s) following a high-energy mechanism were significantly younger than those patients who suffered a low-energy fall (F=14.56, P<0.001).

**Table 1:** Fracture Groups by Mechanism of Injury Percentage of fracture groups by the mechanism of injury

	C1 (n=17)	C2 (n=91)	Combined (n=30)
Low-Energy Fall	47.1 (8)	61.5 (56)	76.7 (23)
Fall Involving Stairs	23.5 (4)	20.9 (19)	20 (6)
High-Energy Fall	29.4 (5)	12.1 (11)	0
Other	0	5.5 (5)	3.3 (1)

Cervical orthoses, namely, Miami-J Collars (Jerome Medical, Moorestown, NJ), were the elected modality of treatment for 105 (76.1%) patients, followed by six patients (5.8%) managed with halo devices. The only patients who underwent operative management of their upper cervical spine fracture(s) had sustained isolated trauma to the axis. Table 2 compares the fracture pattern and the proportion of patients managed with available treatment methods. A proportion of each cohort has been recorded as “other” regarding their management. These were cases in which it was unclear from medical records how their cervical spine injury was managed, or they did not tolerate the treatment modality, largely, Miami-J collars, and therefore it was removed but at an indeterminate time.

**Table 2:** Treatment Modality Percentage of fracture groups by treatment modality

	C1 (n=17)	C2 (n=91)	Combined (n=30)
Miami-J Collar	70.6 (12)	75.8 (69)	80 (24)
Halo	11.8 (2)	4.4 (4)	6.7 (2)
Operative	0	5.5 (5)	0
Other	17.6 (3)	14.3 (13)	13.3 (4)

**Figure 1:**
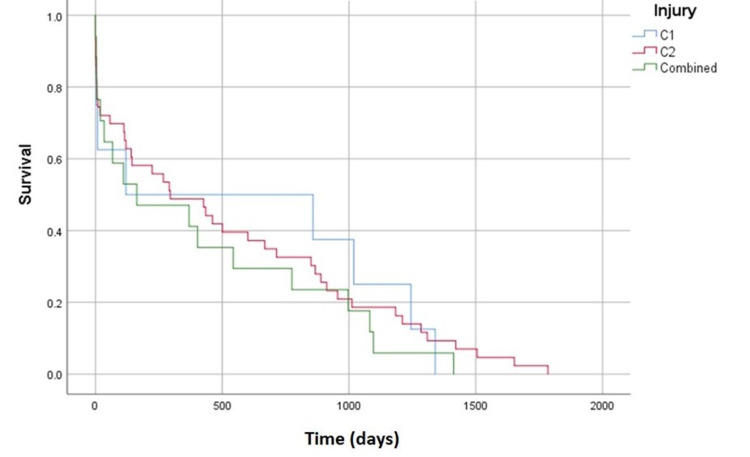
Overall Survivorship Curve Kaplan-Meier survivorship curve for overall mortality in the follow-up period

The overall survival analysis is shown in Figure 1 and demonstrates no statistically significant difference in survivorship between the three patient cohorts (p = 0.658). Nevertheless, the mean time to death in the combined fracture group did tend to be shorter (416 days) than that of isolated fractures of the atlas or axis (574 and 522 days, respectively), as shown in Table 3. No significant difference in time to death was found between each group in a one-way ANOVA (F= 3.22, p=0.726). Similarly, there was no statistically significant difference in the 120-day mortality between the three patient cohorts (p = 0.528), as shown in Table 4.

**Table 3:** Cohort Survivorship Survivorship per fracture cohort and mean time to death

	C1 (n=17)	C 2 (n=91)	Combined (n=30)
% died in the follow-up period	47.1 (8)	47.3 (43)	56.7 (17)
Mean time to death (days)	574	522	416

**Table 4:** Mortality at 120 Days 120-day mortality by fracture cohort

	C1 (n=17)	C2 (n=91)	Combined (n=30)
% of overall mortality occurring within 120 days	50	37.2	47.1
Number died within 120 days	4	16	8

**Figure 2:**
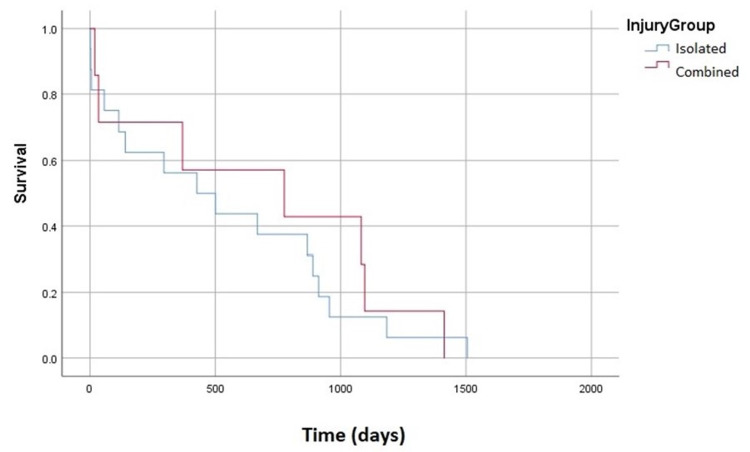
Survivorship Curve of Patients With Cognitive Impairment Comparison of survivorship between isolated and combined fractures in patients with cognitive impairment (p = 0.368)

**Figure 3:**
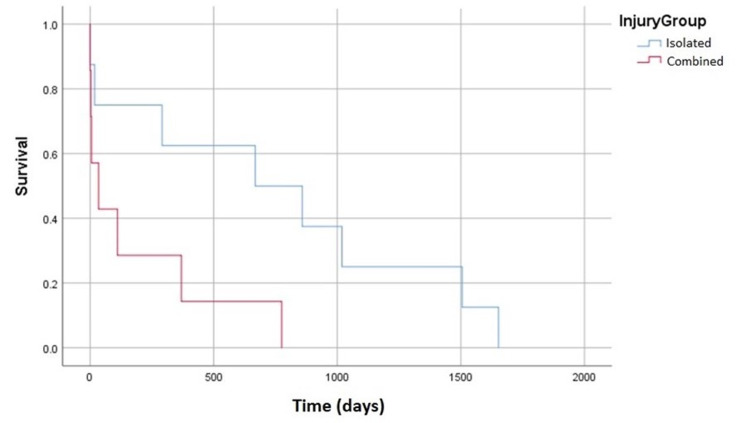
Survivorship Curve of Patients With Previous Fragility Fractures Comparison of survivorship between isolated and combined fractures in patients with previous fragility fractures (p = 0.521)

We continued our analysis into patients who had a diagnosis of dementia prior to their injury. There was no statistically significant difference in survivorship between patients with an isolated atlas or axis fractures and those with combined fractures (p = 0.368). This is shown in Figure 2. A similar analysis was conducted for patients with previous fragility fractures. Patients with isolated and combined fractures had comparable curves (p = 0.521), however, the combined fracture cohort did trend to have shorter survivorship as seen in Figure 3.

## Discussion

This study presents a three-year, case-reviewed data collection of patients with fractures of the atlas and/or axis with an average of nearly four years of follow-up. The main findings are that patients with combined fractures of the upper cervical vertebrae appear to have comparable survivorship to those with fractures of either individual vertebra but that patients who present with combined fractures are significantly older than patients with fractures of either singular vertebra.

Incidence

NHS Greater Glasgow and Clyde is Scotland’s largest health board and serves a population of over 1.14 million [12]. Combined fractures of the atlas and axis complex are a more common injury than previously suggested. We report that 22% of patients with a fracture of the atlas or axis will have a concurrent fracture in the other cervical vertebrae. This overall incidence is comparable to figures in other published literature. Ryan and Henderson, in 1992, reviewed 717 spine fractures and reported that 15% of odontoid fractures and 9% of Hangman’s fractures had combination atlas and axis fractures [13]. In 1997, Greene et al. commented on 340 fractures of the axis and noted that 48 had concurrent fractures of the atlas for an incidence of 14%. A 14-year review of upper cervical spine injuries was performed in 2000 by Gleizes et al. They reported that 31 patients with an atlas fracture had an associated axis fracture. This combined injury was noted in 27% of all upper cervical spine injuries and formed 4% of the total cervical spine fractures [14]. Of note, 64% of patients in their series with a fracture of the atlas had a concurrent fracture of the axis. This figure is generally higher than previously reported [6,13,15-16]. This, in part, can be explained by our inclusion of all fractures of the axis rather than specific fracture patterns to the odontoid peg.

Age distribution

Our data demonstrate that patients with combined fractures are significantly older than patients with fractures of either individual vertebrae. Several studies have reported that the most common cause of a cervical spine fracture in the elderly is a simple fall [9,17-20]. This is a finding that our data supports. Sixty-three per cent of the upper cervical spine injuries included in this review were sustained following a simple fall from standing or sitting. The correlation between the fracture pattern and age is reflected in the causative mechanism of injury. The proportion of patients sustaining their fractures following a simple fall was higher in the generally older, combined fracture group when compared to the younger, isolated atlas fracture cohort. Similarly, the proportion of patients sustaining fractures from a high-energy mechanism was higher in the younger, isolated atlas fracture cohort.

Matthiessen and Robinson reported a similar bimodal age distribution following their national registry-based cohort study of atlas fractures in 2015 [10]. An initial, small peak was observed at 24 years old in which most patients did not have a concurrent axis fracture and a second, larger peak was seen at 81 years old. This second peak revealed a higher incidence of combined fractures in the elderly. These studies demonstrate that measures taken to prevent further rises in the incidence of upper cervical spine fractures should include osteoporosis management and falls prevention targeted at the elderly.

Treatment modality

Since Jefferson’s work in 1920, there have been many studies aimed at determining the most appropriate management of upper cervical spine fractures and most of these include some mention of combined fractures [2]. None of the combined fracture groups was managed operatively from our review. This contrasts with 41.9% of the combined lesions managed operatively as reported in the paper by Gleizes et al. [14].

Mortality

The mortality analysis using the Kaplan-Meier method revealed that patients with combined fractures had comparable mean survival to patients with fractures of either individual vertebrae. These figures contradict work from the late 20th century, which suggested that a combined fracture would predispose a patient to shorter survivorship [3-6]. Conversely, Matthiessen and Robinson’s work did not identify, using Cox regression analysis, atlas fractures as an independent risk factor when they identified patients with atlas fractures having shorter mean survival to those with spinal fractures. They did, however, state that the coincidence of an axis fracture reduced mortality. The postulated reasoning for this was that a combined fracture was more common in the elderly population and this cohort of patients sustain their spinal injury following low-energy trauma, which inherently has lower mortality than those younger patients, sustaining their injuries from higher energy mechanisms [10].

We acknowledge that this study has limitations and possible flaws in the data set. One key limitation is a loss to follow-up, and this is inherently due to our review being of scanned notes online and of communications between general practitioners in the primary care and hospital specialities. There is a potential for patients to have moved out of NHS Greater Glasgow and Clyde’s catchment area and therefore, complications may not appear in our system. Whilst deaths occurring elsewhere in Scotland will be captured on the system, if a patient moved and died elsewhere in the UK, outside Scotland, this would not be updated on our system. There are also limitations in the process of identifying patients with cervical spine fractures due to the fact we only reviewed one imaging modality. Whilst it would be considered the standard practice to obtain a CT scan if an abnormality was demonstrated on a plain film X-ray, it is possible that this may not be felt clinically necessary or indeed likely to change the management plan and thus such a patient would have been missed from our identification.

From our experience, we recommend further work reviewing morbidity and mortality following fractures of atlas and axis in a larger population group and in populations with greater incidences of high-energy traumatic injuries likely to predispose to such fractures. Similarly, reviewing outcomes in areas whereby treatment modality varies to a greater degree would be of benefit to aid in the comparison of operative and nonoperative treatment plans.

## Conclusions

Patients with combined fractures of the atlas and axis and those with isolated fractures of the axis were found to be significantly older than patients with isolated atlas fractures. There is a growing body of evidence that with the increasing proportion of the developed world’s population being elderly, the incidence of upper cervical spine fractures will rise. This is particularly pertinent with regards to combined fractures of the atlas and axis, as these patients are likely to be older and could significantly benefit from targeted preventative measures. Our data shows that the 120-day mortality is proportionally higher in the combined fractures group, however, no long-term statistically significant difference was demonstrated. This evidence contests the notion that combined fractures of the atlas and axis have higher mortality than isolated injuries of either cervical vertebrae.
